# Amantadine-based combination therapy of Parkinson’s disease to prevent fluctuation and dyskinesia – experiences from a Parkinson outpatient clinic

**DOI:** 10.3389/fnagi.2026.1724003

**Published:** 2026-03-26

**Authors:** Christian Oehlwein, Lucas Netzel, Katrin Mittmann, Marita Oehlwein, Walter J. Schulz-Schaeffer

**Affiliations:** 1 Parkinson's Disease Outpatient Clinic, Gera, Germany; 2 Department of Neuropathology, University of the Saarland, Homburg/Saar, Germany

**Keywords:** amantadine, dopamine agonist, dyskinesia, fluctuation, levodopa, MAO type B-inhibitor

## Abstract

**Background:**

The standard therapy of Parkinson’s disease consists in supplementing the depleted dopamine with oral levodopa. The disadvantages of the levodopa monotherapy – comparable to other dopaminergic substances – are the dose-dependent development of dyskinesia in ON-phases, and bradykinesia and akinesia in OFF-phases, the latter being a sign of dopaminergic deficiency with a recurrence of Parkinson’s symptoms.

**Aim of the study:**

In order to omit the side effects of levodopa, we devised a combination therapy consisting of amantadine, a monoamine oxidase type B-inhibitor and a dopamine agonist. Levodopa was added at a low dose, if necessary.

**Methods:**

In a retrospective, monocenter study based on the patient records, we report the long-term results in 132 PD patients who had been treated with the amantadine-based therapy for up to 13 years in an outpatient Parkinson clinic.

**Results:**

In 132 patients with the amantadine-based combination therapy, the mean UPDRS III score improved by 5.7 points from baseline during the first 2 years of treatment. Over the following 3 years it increased and remained at a plateau level slightly below baseline for the next 5 years. At no time did more than 20% of the patients suffer from Hoehn & Yahr stage 3 or higher. Only seven patients exhibited “OFF” periods and only seven (6 with additional levodopa) presented with dyskinesia at any time during therapy. The most important adverse effect was lower leg edema.

**Conclusion:**

The combination therapy of amantadine, a monoamine oxidase type B-inhibitor and a dopamine agonist is an alternate therapeutic approach that may be able to prevent dyskinesia and fluctuations by drug pharmacokinetics and synergistic effects, and may postpone or reduce the amount of levodopa. Prospective controlled studies are needed to compare the effects of the amantadine-based combination therapy with the standard levodopa therapy for Parkinson’s disease.

## 1 Introduction

Since the 1970s, the standard therapy of PD has consisted in supplementing the depleted dopamine with oral levodopa. Dopamine is the main neurotransmitter in the nigrostriatal system, which is crucial for regulating and coordinating movements. The dopamine depletion is related to a pre-synaptic aggregation of the synaptic protein *α*-synuclein (Kramer and Schulz-Schaeffer, 2007; Schulz-Schaeffer, 2010) and occurs together with disturbances in synaptic vesicle cycling (Scott and Roy, 2012). In consequence, the disruption of neurotransmission is associated with the symptoms of neurodegeneration (Kramer and Schulz-Schaeffer, 2007; Schulz-Schaeffer, 2010).

The disadvantages of treatment with levodopa are its short pharmacological half-life and the development of levodopa associated fluctuation and dyskinesia. This forces patients to take their medication at even shorter intervals, up to more than six times per day. The higher the dose of the levodopa medication, the more fluctuating are the blood levels. The patients develop so-called “ON” and “OFF”-phases with dyskinesia during ON-phases. Dyskinesia develops in 16 to 20% of patients after only 9 months of treatment (Fahn et al., 2004) and affect 50 to 60% of patients after 3 to 4 years of treatment (Stocchi et al., 2010; Olanow et al., 2013; Olanow and Stocchi, 2018). Long-term studies indicate that adverse motor effects develop over time in almost all patients treated with a levodopa standard therapy (Olanow and Stocchi, 2018; Ahlskog and Muenter, 2001; Hely et al., 2005).

There have been several attempts to minimize the adverse effects of long-term oral levodopa medication, for example, sustained-release or extended-release levodopa formulations or by administering levodopa continuously with a duodenal pump, by infusing the dopamine agonist apomorphine subcutaneously, or by deep brain stimulation of the subthalamic nucleus (Kalia and Lang, 2015).

An alternative approach is to combine synergistically acting drugs, which allows the levodopa dose to be reduced or its first administration as part of the therapy to be delayed. Birkmayer, who introduced levodopa into the therapy of PD, claimed that levodopa should be used at the lowest possible dose to prevent adverse effects. To keep levodopa doses low, he used synergistically acting drugs and combined low dose levodopa with a monoamine oxidase type B-inhibitor (MAO-B inhibitor) and a dopamine agonist for long-term medication (Birkmayer et al., 1977; Riederer et al., 1983; Birkmayer, 1987). A synergistic effect with a reduction of the “OFF”-periods was observed when combining benserazide with the MAO-B-inhibitor l-deprenyl (Birkmayer et al., 1975). In experimental studies, each of the three drug classes, that is, MAO-B inhibitors, dopamine agonists and NMDA receptor antagonists have shown neuroprotective effects (Szökő et al., 2018; Schapira, 2002; Hallett and Standaert, 2004). Unfortunately, it was not possible to demonstrate the neuroprotective or disease-modifying effects in clinical studies in which the drugs were either used alone, or used individually in combination with levodopa (Löhle and Reichmann, 2010). Prospective studies using all three potentially neuroprotective or disease-modifying drugs in combination are lacking. With its unique pharmacological profile by combining dopaminergic and glutamatergic properties, which account for its dual effect on parkinsonian signs and symptoms and levodopa-induced dyskinesias, amantadine is experiencing a resurgence in Parkinson therapy 50 years after its discovery (Rascol et al., 2021). Furthermore, amantadine has additional and less well-defined pharmacological effects, including on anticholinergic and serotonergic activity.

The aim of the present report is to demonstrate whether the drug combination of amantadine, an MAO-B inhibitor and a dopamine agonist prevent fluctuation and dyskinesia in PD patients and allow lower levodopa concentrations. We report the long-term results in 132 PD patients, treated with an amantadine-based combination therapy as first line therapy over a period of up to 13 years.

## 2 Methods

The patient data were retrieved from the database of a specialist outpatient clinic with a focus on movement disorders, where the patients’ records were administered using the software “ProMed” (ProMed Software GmbH, Bebra, Germany). Patients with the diagnosis “Morbus Parkinson” were selected and were filtered according to the following inclusion criteria:

- fulfilled the diagnosis Parkinson’s disease based on the criteria of the UK Brain Bank (Hughes et al., 1992) and was supported, if available, by a DAT-SPECT;
- had baseline data, medication data and UPDRS III data of a disease course lasting at least 3 years after baseline;
- had received all three components of the combination therapy up to the third year after baseline;
- showed a disease course under the combination therapy for at least 1 year;
- did not have deep brain stimulation;
- had not received infusion or injection therapies (e.g., apomorphine, levodopa/carbidopa intestine gel infusion).

Exclusion criteria were as follows:

- the diagnosis of atypical Parkinson syndromes during the observation period, i.e., atypical parkinsonism or other movement disorders;
- UPDRS III not evaluable because of co-morbidity.

### 2.1 Collected data

The demographic data were sex, age at diagnosis, age at first medication, body height, body weight, date of diagnosis, date of therapy begin, years of observation, date of drop out, reason for drop out. The clinical disease course was quantified using the UPDRS I-IV and the total sum score, the Hoehn & Yahr score and the Schwab-England score.

The core data of the UPDRS (III) and the medication must have been available at least once within the 2 years of follow-up after year 3. Missing values were not extrapolated. In most patients, the complete UPDRS data were available. The UPDRS I, II and IV is missing in two patients in year 2 and in one patient in year 3.

The recorded medication data were as follows: name of preparation/active ingredient and dose of the monoamine oxidase type B-inhibitor, the NMDA-antagonist and the dopamine agonist, as well as the levodopa dose in normal and retarded preparations or as medication on demand, other Parkinson medication, neuroleptics, drugs for sleeping, anti-dementia drugs, antiemetics, and notes about the adverse effects of medication.

### 2.2 Therapeutical procedure

Amantadine (gradually increasing the dose up to 300 mg over 2 weeks) and an MAO type B-inhibitor, i.e., rasagiline 1 mg, or selegiline 7.5 mg form the basis of the therapy. The dopamine agonist is the third component, which is added in a low dose, e.g., pergolide 0.25 mg, or ropinirole 0.5 mg, or pramipexole 0.25 mg, or piribedil 50 mg, or cabergoline 0.5 mg, or a rotigotine 2 mg patch. The dose of the dopamine agonist of the combination therapy is varied depending on the severity of the Parkinson symptoms. At the beginning of the therapy, the patient’s symptoms should be treated optimally and UPDRS I, II, and III scores should be equal to or better than 0, 8, and 15, respectively. The total UPDRS scores of the patients should ultimately not exceed 30 over a long period of treatment. Patients were under regular echocardiographic control.

### 2.3 Data analysis

All data were collected by the personnel of the outpatient clinic Gera. The data sets were extracted from the records by an independent physician (LN). The data were analyzed by a professional medical statistician (KM) in a descriptive manner and were evaluated with SPSS. We evaluated the statistical parameters: mean, median, minimum, maximum, percentiles, standard deviation and calculated frequency tables, and for some items Kaplan–Meier curves and graphical presentations.

### 2.4 Ethics approval

All data were fully pseudonymized, before they were analyzed. The study was approved by the ethics committee of the College of Physicians of the state Thüringen under the number 20558/2014/59. Because this is a retrospective study, the ethics committee did not request an informed consent from the patients to use data from their medical records for research.

## 3 Results

### 3.1 Description of the patient cohort

In total, 132 patients met the criteria; 77 men and 55 women (ratio 1.4:1). They had observed the first symptoms of PD at a mean age of 60.7 years (SD 9.9 years) and were diagnosed with PD at a mean age of 62.6 years (SD 9.7 years). They received their first PD-therapy at a mean age of 63.4 years (SD 9.1 years).

### 3.2 Baseline data

At baseline, 94 of the patients in the study were being treated for the first time, while 38 already had received PD medication previously. Of these, 14 had taken levodopa with a mean dose of 182 mg per day (median 200 mg; range 50–300 mg).

The mean baseline UPDRS total score was 24 (median 21), with a mean score of 16.1 for the UPDRS part III (motoric items; median 14). One patient exhibited dyskinesia (items 32–34), three patients had early morning dystonia (item 35), and two patients had OFF-periods (items 36–39). Orthostatic disturbances were recorded in three patients; 59 patients suffered from depressive symptoms (item 3), and 17 patients from sleep disturbances (item 41).

The mean Hoehn & Yahr score (130 data sets) was 1.7, but 116 patients scored maximum 2.0 (postural stability). The mean Schwab-England score was 89.0% (129 data sets), with 122 patients reaching at least the 80% level (usually completely independent) and 67.5 the 90% level (completely independent).

### 3.3 Follow-up

The combination therapy was completed by drugs of all three substance classes in all patients within the first 3 years after the baseline measurements starting with a monoamine oxidase type B inhibitor, followed by an NMDA-antagonist and a dopamine agonist by 80 patients in year 1, 37 in year 2, and 15 in year 3.

In the fifth year, the doses of the MAO type B inhibitors were 1 mg rasagiline or 7.5 mg selegiline (mean 8.1 mg). Those of the NMDA-antagonists were 225 mg amantadine (mean 211.5 mg) or 20 mg budipine (range 10–60 mg). The dopamine agonist was administered at approximately 70% of the maximal dose. Fourteen of the 90 patients received two dopamine agonists. An overview of the most common medication at baseline and in the years 2, 5 and 8 is given in Table 1. Levodopa was added in 58 of the 118 levodopa naïve patients at an average of 7.6 years (median 7.0 years) after beginning the PD-therapy. Five years after starting the PD therapy, 32% of the patients were receiving an average daily dose of 237.5 mg levodopa (median 250 mg, range 50 – 450 mg).

**Table 1:** Overview about clinical course and medication.

Item	Baseline *n* = 132	Year 2 *n* = 132	Year 5 *n* = 90	Year 8 *n* = 30
UPDRS total, mean ± SD (median; range)	24.0 ± 12.6 (21; 2–66)	16.1 ± 7.8 (15; 3–41)	22.9 ± 12.9 (22; 2–65)	22.8 ± 12.9 (19.5; 5–54)
UPDRS II, mean ± SD (median; range)	6.5 ± 3.4 (6; 0–17)	5.1 ± 2.5 (5; 1–11)	6.9 ± 3.9 (7; 0–21)	6.7 ± 3.6 (6; 1–36)
UPDRS III, mean ± SD (median; range)	16.1 ± 9.0 (14; 1–47)	10.4 ± 5.5 (9; 2–27)	14.8 ± 8.6 (14; 2–42)	14.9 ± 8.5 (13; 4–36)
MAO-B inhibitor mean (median)	*N* = 123 without MAO-B inhibitor	Rasagiline 1 mg (1 mg) *n* = 74 or selegiline 7.68 mg (7.5 mg) *n* = 53	Rasagiline 1 mg (1 mg) *n* = 48 or selegiline 8.06 mg (7.5 mg) *n* = 40	Rasagiline 1 mg (1 mg) *n* = 11 or selegiline 7.5 mg (7.5 mg) *n* = 17
Dopamine agonist Mean (median) Most frequent treatment regimes described	*N* = 109 without DA	Pramipexole 2.38 mg (2.25 mg) *n* = 51 or ropinirole 9.52 mg (9.0 mg) *n* = 27 or cabergoline 5.43 mg (6.0 mg) *n* = 7 or pergolide 3.375 mg (3.375 mg) *n* = 6 or rotigotine 8.91 mg (10.0 mg) *n* = 11	Pramipexole 3.33 mg (3.0 mg) *n* = 38 or Ropinirole 12.86 mg (14.0 mg) *n* = 25 or Cabergoline 7.78 mg (7.5 mg) *n* = 14 or Pergolide 4.43 mg (4.25 mg) *n* = 7	Pramipexole 3.11 mg (3.0 mg) *n* = 13 or Ropinirole 16.0 mg (16.5 mg) *n* = 6 or Cabergoline 6.83 mg (6.5 mg) *n* = 6 or Pergolide 3.25 mg (2.75 mg) *n* = 3
NMDA-antagonist Mean (median)	*N* = 119 without NMDA-antagonist	Amantadine 218.75 mg (225 mg) *n* = 88 Budipine 20.25 mg (20.0 mg) *n* = 40	Amantadine 211.5 mg (225 mg) *n* = 65* Budipine 22.5 mg (20.0 mg) *n* = 32*	Amantadine 204.5 mg (225 mg) *n* = 22* Budipine 26.36 mg (30.0 mg) *n* = 11*
Levodopa mean (median, range)	*N* = 14 with levodopa	229 mg (250 mg, 50–500 mg) *n* = 13	237.5 mg (250 mg, 50–450 mg) *n* = 28	298 mg (300 mg, 150–500 mg) *n* = 11

Missing data were not interpolated from previous or later medication plans. *Changes of treatment during the observation period result in double counting.

The levodopa equivalent daily dose was calculated according to Jost et al. (2023). In the fifth year, the mean LEDD was 727.94 ± 275.92, a median of 658.34, a minimum of 270 and a maximum of 1138.34. In the eighth year, the mean LEDD was 792.58 ± 271.81, a median of 770.02, a minimum of 325 and a maximum of 1345.84.

**Figure 1:**
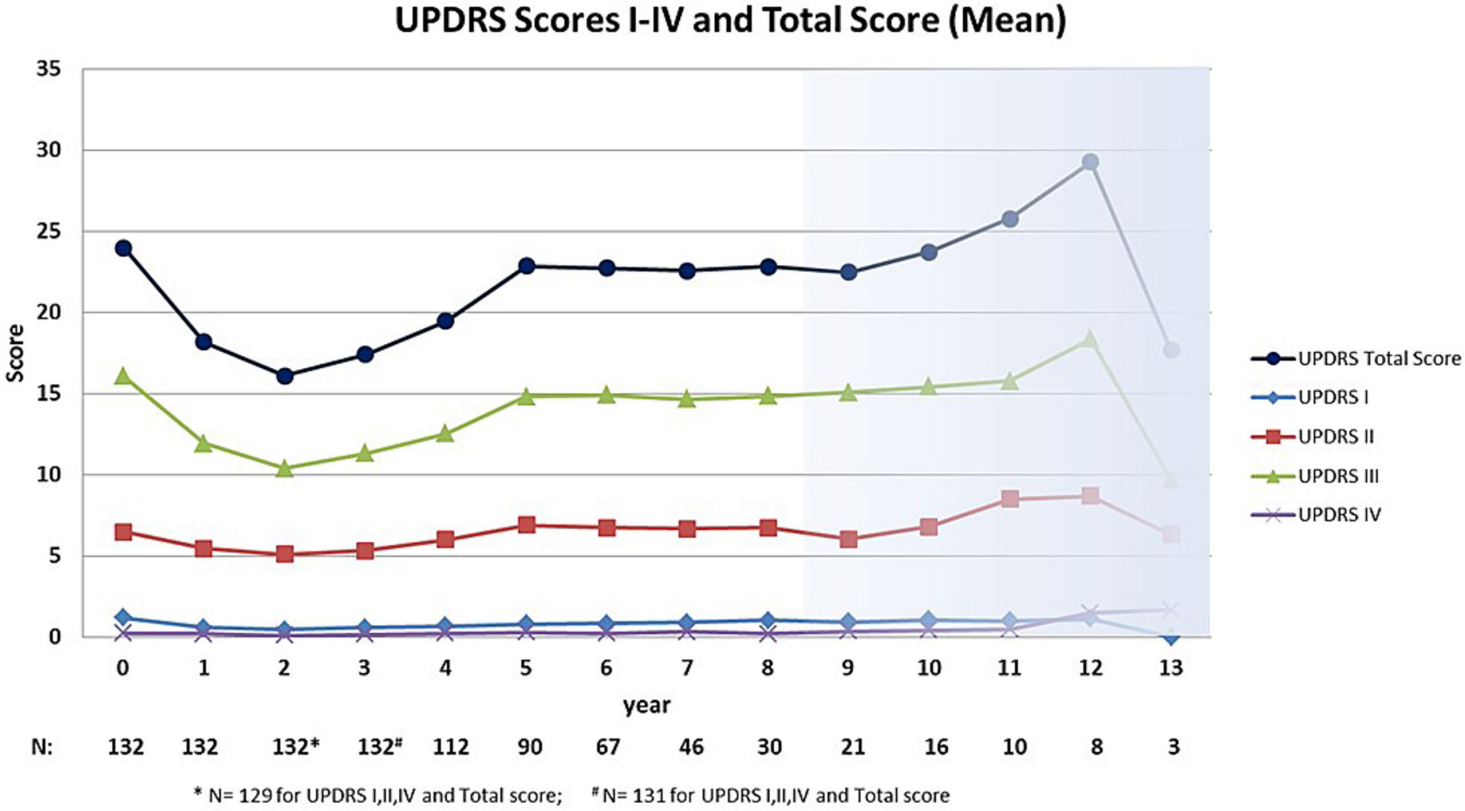
UPDRS scores of the amantadine-based combination therapy patients.

The curves of the mean UPDRS total score and the UPDRS III score had the same shape (Figure 1). During the first 2 years of treatment, the total score improved by an average of 7.9 points (median 6 points) and the UPDRS III score by 5.7 points (median 3.5). Over the following 3 years they increased to a plateau slightly lower than the baseline score, where they remained for the next 5 years. The baseline was exceeded after an average of 11 years. The main contribution to the total score came from sub-scores III (motor items) and II (activities of daily living). UPDRS II showed a relatively stable course with a mean variation of about 1.5 points over 10 years. UPDRS I and UPDRS IV did not contribute substantially to the total score (Figure 1). The shape of the UPDRS score curves of the subgroups “*de novo*” and “pretreated” patients did not differ notably from those of the entire group.

**Figure 2:**
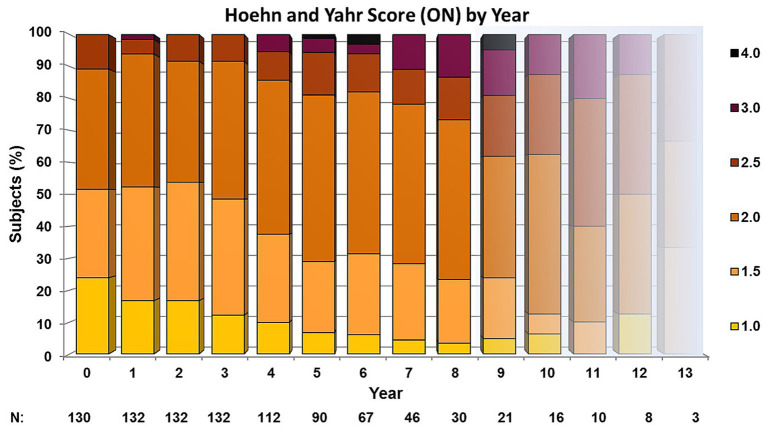
Hoehn and Yahr scores of the amantadine-based combination therapy patients.

The Hoehn & Yahr scores showed a stable distribution for the first 3 years (Figure 2). The percentage of patients with a score of 2.5 or higher increased from 14 to 27% in the years 4 to 8 and further increased in these stages from 38 to 60% in the following years. At no point in the study, did more than 20% of the patients suffer from a Hoehn & Yahr stage 3 or higher.

The Schwab-England ADL scores also showed a stable distribution for the first 3 years. From year 4 to year 8, the percentage of patients with a score of 90% and higher, meaning that the patients were completely independent, decreased from 57 to 47%. The number of patients with a score of 70% (mostly independent) and lower increased in these years from 4.5% in year 4 to 16.6% in year 8, and only one patient scored 50% (mostly dependent).

### 3.4 Adverse effects of the amantadine-based combination therapy (including those of levodopa) during the observation period

During the course of the disease, seven patients experienced dyskinesia (Table 2), all but one in combination with levodopa (UPDRS items 32–34). Twenty-five patients had early morning dystonia (item 35), and seven patients had OFF-periods (items 36–39). Orthostatic disturbances were recorded in six patients (item 42). Fifty-six patients suffered from depressive symptoms (item 3).

**Table 2:** Therapy-related events during the amantadine-based combination therapy, as noted in the patient records.

Adverse event	Number of affected patients	Comment, possible cause, treatment
Edema (lower leg)	52	Diuretica, lymph drainage, support stockings.
Sleep disorder	41	Trazodone at night may be indicated.
Early morning dystonia	25	
Daytime sleepiness	21	Related to sleep disorder.
Nausea	19	Mostly related to discontinuation of domperidon.
Hallucinations	15	
Dry mouth	14	Problem of budipine medication.
Dizziness	11	
Skin reaction	9	Six were reactions to patches.
Impulse control disorder	8	4x hypersexuality, 2x binge eating, 2x punding, 1x compulsive shopping.
Dyskinesia	7	6 in combination with levodopa
Off-periods	7	
Orthostatic disturbances	6	
Relative QT time >120%	4	Budipine dose to be adjusted.
Heartburn	4	
Nightmare	2	
Livedo reticularis	2	Adverse event of amantadine medication.
Hypotension	2	
Head pressure	2	
Anxiety	2	

In the fifth year, two patients exhibited dyskinesia (items 32–34), nine patients early morning dystonia (item 35), and two patients had OFF-periods (items 36–39). No patients suffered from orthostatic disturbances (item 42). Nineteen patients suffered from depressive symptoms (item 3).

During the course of the disease, eight patients exhibited impulse control disorders. Hallucinations were reported in 15 patients, and a psychosis was diagnosed in two. Four additional patients reported a feeling/sense of presence.

The main adverse effects were edema (lower legs, 52 patients) and sleep disorders (41 patients; UPDRS item 41). While sleep disorders, possibly together with daytime somnolence (21 patients), are an immediate effect of the stimulation of the dopaminergic system, edema usually occurs later in the course of the disease.

### 3.5 Drop outs

**Figure 3:**
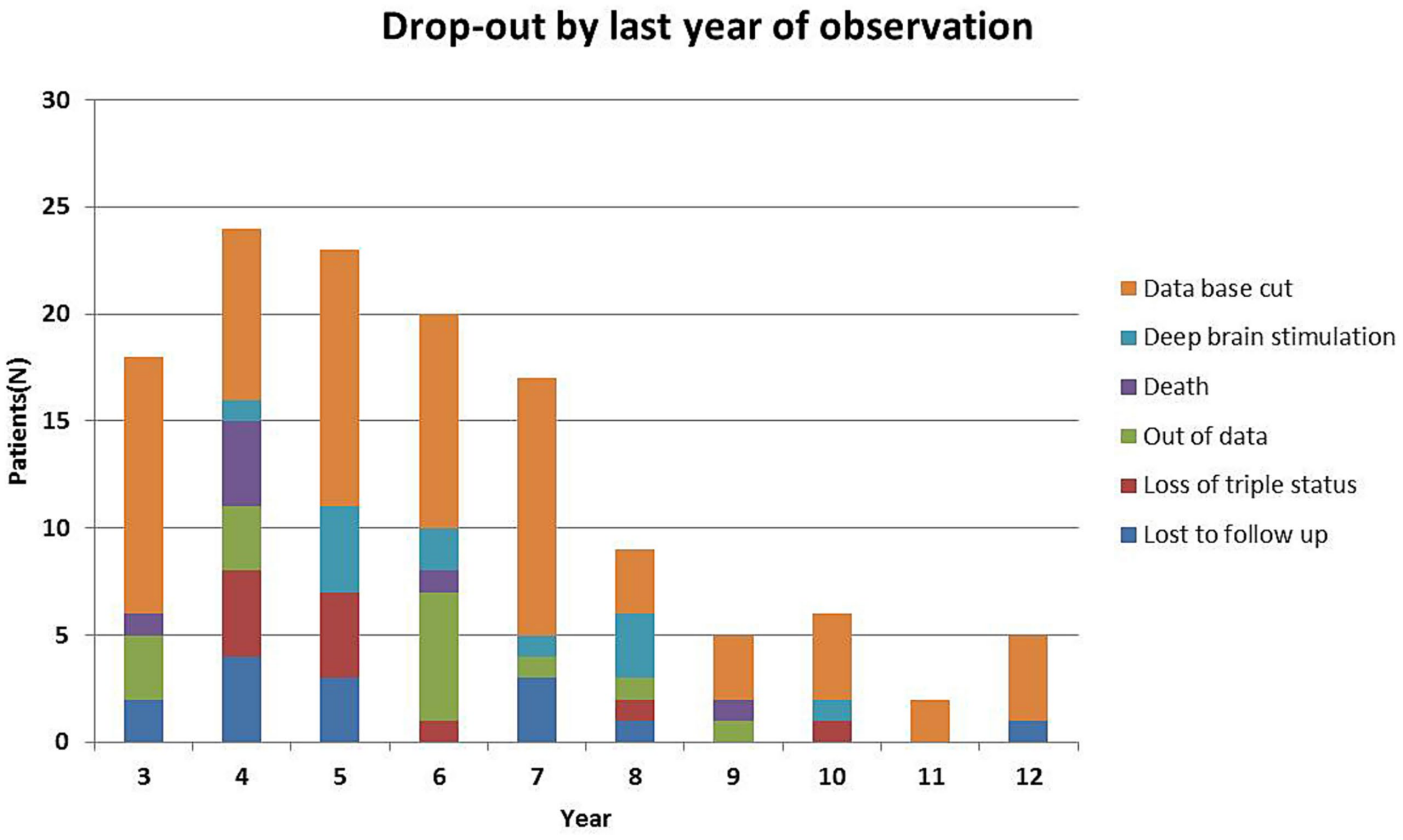
Reasons for patient’s drop-out during the observation period.

Figure 3 shows the number of study dropouts per year. Database cut counts those patients that are still under observation, but not long enough in the study to have more data, because the patients were included consecutively. Patients out of data, loss to follow up and death are common reasons for dropouts. A drop out because of deep brain stimulation (*n* = 12) may cause a bias. Deep brain stimulation was offered to patients at an early stage of the disease to prevent quitting work or hobby activities. In 11 patients, the amantadine-based combination therapy was changed to a levodopa-based combination therapy because of hallucinations (*n* = 5), edema (*n* = 3), and in one patient each because of impulse control disorder, surgical complications, or exsiccation.

## 4 Discussion

Long-term follow-up studies of levodopa-based Parkinson therapies have revealed three clinical phases of the therapy, the honey moon phase, a steady state at a level near the baseline and the progression phase (Barbeau, 1976; Garcia Ruiz et al., 2004; Cabo Lopez et al., 2010; Reinoso et al., 2015). PD patients treated with the amantadine-based therapy go through the same three phases of the disease course as those with levodopa therapy, but with markedly longer durations of the phases of improvement and steady state (Figure 1). It might be that the delayed disease progression under the amantadine-based therapy reflects the postulated neuroprotective effects of monoamine oxidase type B-inhibitor, NMDA antagonist, and dopamine agonist (Riederer et al., 2002). This may become relevant for the course of the disease, when the drugs are given in combination, similar to the combination of selegiline with levodopa in comparison with monotherapies using each component (Palhagen et al., 2006; Palhagen et al., 1998).

The main advantage of the amantadine-based therapy is that the administered drugs have long plasma half-lives. This avoids rapid increases and decreases of the blood levels of the drugs, which is instrumental in preventing ON- and OFF-periods. A second advantage is the synergistic effect of the drug combination (Greenamyre and O'Brien, 1991). All three drugs contribute to a constantly elevated dopamine level. As a consequence, “ON”- and “OFF”-periods only occur in PD patients as a rare adverse effect when levodopa is added. The only period of under-medication that is seen in individual patients, is an early morning “OFF”-period (early morning dystonia, UPDRS item 35).

### 4.1 Comparison of the amantadine-based therapy with those of other studies

Reinoso et al. (2015) investigated the rate of progression of motor symptoms in 576 PD patients whose data was derived from the movement disorders database of the National Neuroscience Institute of Singapore from 2002 to 2012. Only those PD-patients were included, whose baseline assessment was performed within 2 years of the diagnosis date and had a follow-up time of at least 3 years. The overall annual UPDRS motor score progression from baseline ranged from 0.62 to 3.67% and patients returned to the baseline score 2–2.5 years after diagnosis (Reinoso et al., 2015). Alves et al. (2005) investigated the rate of progression of motor symptoms in a population-based cohort of 232 PD patients with a mean age of onset of 62 years. The patients were followed prospectively over an 8-year period by examinations 4 and 8 years after baseline. The mean levodopa doses were 490 mg/d at baseline, 626 mg/d at the 4-year follow-up and 640 mg/d at the 8-year follow-up. The patients showed an UPDRS motor score progression with an annual worsening of 3.3 points (3.1%) over 8 years and an annual Hoehn & Yahr score progression of 0.16 points (3.2%). Over a mean period of 6.36 years, Jankovic and Kapadia (2001) reported an annual rate of decrease of the UPDSR motor part of 1.38 points in the “ON” and 1.58 points in the “OFF” state of best medical treated PD patients. Louis et al. (1999) reported an increase of the UPDRS motor score at an annual rate of 1.5 points (1.5%) in a community-based cohort of 237 PD patients from Manhattan, NY, of whom 70% received levodopa. This rate increased to 3.6 points in those patients who died during the next follow-up period. The Swedish Parkinson Study Group reported the results of a two-phase study of a 7-year observation period using an MAO-B inhibitor (selegiline) versus placebo in the initial phase of *de novo* PD patients, and selegiline + levodopa versus levodopa alone in the second phase. Patients treated with selegiline and later with levodopa reached the initial baseline at 36 months of the second phase (≈ 4 years from the beginning of the study) and crossed the baseline at 60 months of phase II, whereas the first phase placebo-, second phase levodopa-patients never went below baseline (Palhagen et al., 2006; Palhagen et al., 1998).

In comparison, the amantadine-based therapy patients generally fell below the baseline of the UPDRS motor score (and the UPDRS total score) at least for the first 8 years (Figure 1) and showed an annual Hoehn & Yahr score progression in the first 8 years of disease of 0.049 points (Figure 2).

### 4.2 Why are dyskinesias and impulse control disorders seldom during the amantadine-based therapy?

Levodopa is known to cause dyskinesia, that is, short-term levodopa-induced dyskinesia (LID), and dopamine agonists may cause impulse control disorders (Kalia and Lang, 2015). The dyskinetic movements are explained with sensitization of glutamatergic NMDA-receptors by the therapeutically induced discontinuous dopamine levels. Dopamine and NMDA receptors act synergistically on medium spiny neurons of the basal ganglia circuit in a way similar to long-term potentiation (Verhagen Metman et al., 2000). Medium spiny neurons integrate input from the dopaminergic nigrostriatal system and the glutamatergic corticostriatal system, the so-called motor circuitry. Non-physiological stimulation of dopamine receptors on striatal medium spiny neurons leads to changes in the subunit phosphorylation of co-expressed NMDA-receptors. This results in an increase in synaptic efficacy and an enhanced sensitivity to the corticostriatal synaptic input (Oh and Chase, 2002). Recent results suggest, that a deprivation of dopaminergic afferents lead to a relocation of postsynaptic D1 receptors, building physical and functional interactions with mGlu5 receptors. D1-mGlu5 heterodimers may mediate a synergistic activation in the presence of both receptor agonists (Sebastianutto et al., 2020).

Impulse control disorders are assumed to be associated with the limbic sub-circuitry, which comprises limbic brain regions including the prefrontal cortex, the amygdala, the nucleus accumbens and the ventral tegmental area. A synaptic change following dopamine deprivation and an alteration of the glutamate homeostasis, resulting in a change of effectiveness of transmission in the neural circuit, is thought to lead to an overstimulation of the limbic circuitry (Kalivas, 2009; Linazasoro, 2009).

Although amantadine (and budipine) are ranked as NMDA receptor antagonists, it becomes evident that their mechanism of action is manifold (Danysz et al., 2021). The anti-dyskinetic effects seem to be mediated by a low-affinity, non-competitive antagonism of the N-methyl-D-aspartate (NMDA)-glutamate receptor subtype at the phencyclidine binding site, which is localized inside and at the sigma 1- binding site localized outside the NMDA receptor gated cation channel (Perez-Lloret and Rascol, 2018; Kornhuber et al., 1991). An additional effect on nicotinic acetylcholine receptors may add to the anti-dyskinetic effects, as alterations in the nicotinic cholinergic system are implicated in the pathological events leading to dyskinesia (Perez et al., 2018). The diversity of effects and the low NMDA receptor binding affinity makes the pharmacological effect more reversible and reduces side effects in the clinical use (Kew and Kemp, 2005; Paoletti and Neyton, 2007). It is assumed that amantadine modulates the sensitization of dysregulated corticostriatal and corticolimbic circuits by blocking NMDA receptor channels (Verhagen Metman et al., 1998). An alternative explanation states that the anti-dyskinetic effect of amantadine is mediated via inhibition of striatal type 2 K + channels (Kir2) (Shen et al., 2020). Shen et al. (2020) suggested a reduction of the disparity in the excitability of direct- and indirect-pathway striatal spiny projection neurons by blocking Kir2 channels via amantadine.

## 5 Limitations

This is a retrospective, monocenter study based on the patient records of a specialized outpatient clinic. Because of the retrospective character of data evaluation, no control group of the same institution was available. Therefore, in the discussion the study results were compared to those published from other PD-studies. A direct comparison of the observational data with the results of the standard levodopa therapy is therefore not possible. Here, a prospective case–control study is necessary. The outpatient clinic that provided the patient records is experienced in clinical studies, and the patients are used to filling out standardized questionnaires. The analysis of the clinical course, however, was based on the UPDRS and the notes made during the patient’s visits. A patient selection was that the outpatient clinic has a focus on Parkinson’s disease. As the study was not blinded the evaluation of the patient data and the supervision of the evaluation was performed by external physicians (LN, WJSS) to minimize a potential observer bias. During the 13 years of observation, the patients were given different medical products of dopamine agonists and NMDA-antagonists. As an alternative to amantadine, budipine was used in tremor-dominant patients up to its withdrawal from the market. Budipine-treated patients got regular electrocardiographic controls to monitor the heart rate-corrected QT interval. A higher risk for cardiac valve fibrosis was reported for cabergoline and pergolide than for other dopamine agonists and all patients were under regular echocardiographic control. Missing data and drop outs may affect results. For minimizing this risk, missing data were not interpolated and the reasons for drop outs were given in the Drop out section. The decline of patient numbers over the years of observation in our study may have an influence on the results but the decline is similar to that in other long-term PD-studies (Reinoso et al., 2015; Alves et al., 2005; Louis et al., 1999). For this reason we shaded the curves of the longest treatment duration (years 9 to 13) in Figures 1 and 2 because the number of patients had markedly decreased by that time and fall under 20% of the baseline numbers, but we did not want to withhold the results.

## 6 Conclusion

Our data indicate that exploiting the effects of a combination of amantadine, a dopamine agonist and a monoamine oxidase type B-inhibitor may help to reduce levodopa side effects. The long plasma half-lives of the administered drugs avoids rapid increases and decreases of the blood levels of the drugs, which is instrumental in preventing ON- and OFF-periods. The administration of amantadine helps to avoid dyskinesia and impulse control disorders. A prospective, controlled study is needed to evaluate the amantadine-based combination therapy and compare it with the standard levodopa therapy.

## Data Availability

The raw data supporting the conclusions of this article will be made available by the authors, without undue reservation.
